# Effects of Hypoxia Exposure on Hepatic Cytochrome P450 1A (CYP1A) Expression in Atlantic Croaker: Molecular Mechanisms of CYP1A Down-Regulation

**DOI:** 10.1371/journal.pone.0040825

**Published:** 2012-07-16

**Authors:** Md. Saydur Rahman, Peter Thomas

**Affiliations:** Marine Science Institute, University of Texas at Austin, Port Aransas, Texas, United States of America; The Scripps Research Institute, United States of America

## Abstract

Hypoxia-inducible factor-α (HIF-α) and cytochrome P450 1A (CYP1A) are biomarkers of environmental exposure to hypoxia and organic xenobiotic chemicals that act through the aryl hydrocarbon receptor, respectively. Many aquatic environments heavily contaminated with organic chemicals, such as harbors, are also hypoxic. Recently, we and other scientists reported HIF-α genes are upregulated by hypoxia exposure in aquatic organisms, but the molecular mechanisms of hypoxia regulation of CYP1A expression have not been investigated in teleost fishes. As a first step in understanding the molecular mechanisms of hypoxia modulation of CYP1A expression in fish, we characterized CYP1A cDNA from croaker liver. Hypoxia exposure (dissolved oxygen, DO: 1.7 mg/L for 2 to 4 weeks) caused significant decreases in hepatic CYP1A mRNA and protein levels compared to CYP1A levels in fish held in normoxic conditions. *In vivo* studies showed that the nitric oxide (NO)-donor, *S*-nitroso-N-acetyl-DL-penicillamine, significantly decreased CYP1A expression in croaker livers, whereas the competitive inhibitor of NO synthase (NOS), *N_ω_*-nitro-L-arginine methyl ester, restored CYP1A mRNA and protein levels in hypoxia-exposed (1.7 mg DO/L for 4 weeks) fish. *In vivo* hypoxia exposure also markedly increased interleukin-1β (IL-1β, a cytokine), HIF-2α mRNA and endothelial NOS (eNOS) protein levels in croaker livers. Pharmacological treatment with vitamin E, an antioxidant, lowered the IL-1β, HIF-2α mRNA and eNOS protein levels in hypoxia-exposed fish and completely reversed the down-regulation of hepatic CYP1A mRNA and protein levels in response to hypoxia exposure. These results suggest that hypoxia-induced down-regulation of CYP1A is due to alterations of NO and oxidant status, and cellular IL-1β and HIF-α levels. Moreover, the present study provides the first evidence of a role for antioxidants in hepatic eNOS and IL-1β regulation in aquatic vertebrates during hypoxic stress.

## Introduction

Cytochromes P450 (CYPs) comprise a large gene superfamily encoding a diverse group of heme-thiolate monooxygenase enzymes that catalyze the oxidation of organic substances [1]–[3]. In the CYPs superfamily, the CYP1 family enzymes are of broad interest because they play a major role in the biotransformation of a variety of endogenous substances such as lipids, steroids, and vitamins [1], [2], [4] and also environmental toxicants, in particular halogenated aromatic hydrocarbons (HAHs), polycyclic aromatic hydrocarbons (PAHs), and polychlorinated biphenyls (PCBs) [5], [6]. Therefore, endogenous and exogenous factors that regulate the expression and activity of CYP1s can also influence the biotransformation and toxicity of these compounds.

CYP1A is the most intensively studied vertebrate CYP1 paralog and comprises two genes CYP1A1 and CYP1A2 in mammals and other tetrapods [7], [8]. Most teleost fishes, on the other hand, have one CYP1A gene [4], although two hybrid CYP1A genes, CYP1A1 and CYP1A2, with 96% sequence identities, have been characterized in rainbow trout [9]. Some regions of the trout CYP1A2 cDNA are identical to mammalian CYP1A1 but differ from CYP1A2 cDNA. These homology patterns suggest that a single CYP1A gene exists in teleost fishes [4], [5].

Aquatic organisms are frequently exposed to environmental hypoxia due to natural seasonal fluctuations in dissolved oxygen and as a result anthropogenic eutrophication [10], [11]. Hypoxia induces a series of adaptive cellular responses including generation of ATP through the glycolytic pathway involving increases in glycogen phosphorylase and aldolase as well as increased production of stress-related proteins [12], [13]. At the molecular level, the adaptation involves increases in mRNA transcription of genes encoding for proteins involved in anaerobic and fat metabolism [13]–[15]. Many of these cellular and molecular responses to hypoxia are likely controlled by hypoxia-inducible factor (HIF), a transcription factor which regulates the expression of numerous genes during exposure to hypoxia [16], [17].

HIF is a heterodimeric transcription factor comprised of an oxygen-sensitive alpha subunit (HIF-α) and an oxygen-insensitive beta subunit (HIF-β; also referred to as the aryl hydrocarbon receptor nuclear translocator, ARNT), both of which belong to the basic helix-loop-helix (bHLH) family of transcription factors [17], [18]. Under normoxic conditions, HIF-α is rapidly degraded by an ubquitination-proteosome pathway initiated by the prolyl hydroxylase enzyme (PHD), preventing it from associating with ARNT to form the transcriptionally active dimer [16]. Under hypoxic conditions, on the other hand, the activity of PHD enzyme is decreased, so that HIF-α is stabilized and dimerizes with ARNT, replacing the chaperone heat shock protein 90 (HSP90) to form the active heterodimer [17], [19], [20]. The active HIF-α complex is a strong activator of transcription and binds to hypoxia response elements in the promoter or enhancer regions of genes resulting in marked increases in their expression. In addition to HIF-α, HIF-β or ARNT has been identified as a dimerization partner for both HIF-α and aryl hydrocarbon receptor (AhR, a bHLH DNA-binding protein) which is activated by various xenobiotic ligands [21]. After binding to ligand, AhR is translocated into the nucleus where it dimerizes with ARNT. The AhR/ARNT complex then binds to the xenobiotic responsive elements in the upstream coding regions of genes and transcriptionally upregulates various xenobiotic-metabolizing genes such as CYP1A [3], [21], [22]. The fact that the HIF-dependent response to hypoxia and the AhR/CYP1A response to xenobiotic chemicals involve the same partner, ARNT, provides a potential site of interactions between these two pathways [23], [24], with possible adverse effects on an organism’s ability to adapt to these environmental stressors.

It is not surprising, therefore, that the xenobiotic-dependent induction of CYP1A gene can be modulated by hypoxia. Recent *in vivo* studies have demonstrated that hypoxia decreases ethoxyresorufin-*O*-deethylase (EROD, an index of CYP1A enzymatic activity; [25]) activity in zebrafish embryos [26] and CYP1A mRNA levels in hepatic tissues of Atlantic cod [27]. Similarly, *in vivo* studies in rabbit liver and *in vitro* studies in rodent hepatocytes have shown that hypoxia decreases CYP1A1 and CYP1A2 mRNA and protein expression, and these down-regulations of CYP1As are mediated through HIF-α, cellular cytokines (CTK) and reactive oxygen species (ROS) [28], [29]. In addition, several lines of evidence indicate that the excessive production of nitric oxide (NO) in mammalian cells is a major factor mediating the suppression of hepatic metabolism and CYP1A1 activity [30], [31].

NO is a highly diffusible and biologically active free radical which is endogenously synthesized by NO synthase (NOS) enzymes during conversion from L-arginine to L-citrulline [32], [33]. NO is recognized to have a key role in virtually all aspects of cellular and molecular physiology and has been implicated in many pathophysiological processes such as inflammation and neurodegeneration [34], [35]. In addition, NO directly reacts with superoxide anions (O_2_.^−^, a potent ROS) to form peroxynitrite (ONOO^−^, a potent oxidant and nitrating agent) which attacks proteins, lipids, and DNA as well as depleting antioxidant defense systems [33], [36].

Indeed, NO was first implicated as a biological signaling molecule because it triggers rapid responses to hypoxia [32], [37]–[39]. There is increasing evidence that NO stabilizes HIF-1α in human cultured cells under nonhypoxic conditions [40]. Similarly, ROS act as oxygen sensors and also appear to play an important role in the upregulation of HIF-1α in cultured human cells [41], [42]. On the other hand, CTK can also increase HIF-1α levels under both nonhypoxic and hypoxic conditions [40]. Moreover, CTK reduce the enzymatic activity of CYP1A by stimulating the generation of ROS and NO [28], [30]. We have recently demonstrated that hypoxia drastically increases hepatic ROS generation and HIF-αs expression in fish livers [43]. On the basis of these results and the findings of mammalian studies it is proposed that hypoxia-induces down-regulation of CYP1A in croaker livers and it is mediated by increases in NO, ROS, CTK and HIF-αs. We have also found that the hypoxia-induced ROS and HIF-αs upregulation are decreased by *in vivo* treatment with an antioxidant [43]. It is therefore hypothesized that the application of antioxidants to hypoxia-stressed fish will reverse hypoxia down-regulation of hepatic CYP1A.

The effects of hypoxia on CYP1A expression were investigated in Atlantic croaker, a relatively hypoxia-tolerant marine teleost that inhibits estuaries along the US Atlantic and Gulf of Mexico coasts which are seasonally hypoxic (dissolved oxygen, DO: <2.0 mg/L; [20]). Three experimental approaches were adopted to clarify the potential mechanisms involved in hypoxia regulation of CYP1A in this teleost model. In the first set of experiments, we characterized Atlantic croaker CYP1A, and examined mRNA levels, microsomal protein expression, and immunohistochemical detection of CYP1A in hepatic tissues in response to different exposure periods (1, 2, and 4 weeks) of hypoxia (1.7 mg DO/L). The effects of a NO-donor and NOS-inhibitor on CYP1A enzymatic activity, mRNA and protein expression in croaker livers were examined in a second series of *in vivo* experiments under both hypoxic and normoxic conditions in order to determine the role of NO in the CYP1A regulation in response to hypoxia. In the final *in vivo* experiments, the effects of treatment with vitamin E on the CYP1A expression in response to hypoxia as well as hypoxia regulation of hepatic interleukin-1β (IL-1β, a CTK; [28]), HIF-α, and endothelial NO synthase (eNOS) were investigated in order to test the effects of antioxidant status on these responses to hypoxia.

## Materials and Methods

### Chemicals

Ethoxyresorufin, bovine serum albumin (BSA), TRI reagent, glucose-6-phosphate dehydrogenase, NADP, NADPH, quinaldine, EGTA and EDTA were obtained from Sigma-Aldrich (St. Louis, MO, USA). Mouse monoclonal anti-CYP1A antibody (C10-7) was generous a gift from Dr. Charles D. Rice, Clemson University, SC, USA. Rabbit polyclonal antibody against eNOS, rabbit anti-actin, and goat polyclonal to mouse IgG horseradish peroxidase (HRP)-linked antibodies were obtained from Santa Cruz Biotechnology (Santa Cruz, CA), Novus Biologicals (Littleton, CO), and Cell Signaling (Danvers, MA), respectively. Oligonucleotides were synthesized by Eurofins MWG Operon (Huntsville, AL). Materials for molecular biology were purchased from Agilent Technologies (La Jolla, CA), Promega (Madison, WI), and Invitrogen (Carlsbad, CA). All other chemicals were obtained from Fisher Scientific (Pittsburgh, PA, USA) unless noted otherwise.

### Experimental Animals

Young (yr 1) Atlantic croaker, *Micropogonias undulatus* (10–11 cm length; 12–18 g body weight, BW), were collected by shrimp trawl in the Aransas Bay, TX, by local fisherman according to the approved wildlife species capture rules and regulations by the Texas Parks and Wildlife (permit no. SPR-0790-184). Fish were then transported to nearby fish holding facilities at the University of Texas Marine Science Institute, treated with Paracide-F (Argent Chemical, Redmond, WA) at 170 ppm in seawater for 1 h to minimize parasite infections, and transferred to large indoor tanks (4,727 L) equipped with a recirculating seawater system (salinity 30–32 ppt) and maintained under control photoperiod (11L:13D) and temperature (22±1°C) conditions. Fish in each tank were fed the same amount of chopped shrimp daily (3% BW/day) and acclimated in fully aerated recirculating seawater to laboratory conditions for at least 1 month prior to experimentation.

### Ethics Statement

All experimental protocols were approved and performed according to the ethical rules by the University of Texas at Austin Institutional Animal Care and Use Committee (IACUC, protocol# 09022701) and followed standard rules by the IACUC on Animal Care guidelines on the use of animals in laboratory research.

### Experiment 1: Effects of Short- (1 Week) and Long-term (2 and 4 Weeks) Hypoxia Exposure on Regulation of CYP1A

A detailed account of the hypoxia-exposure methods used in this study has been described previously [44]. Briefly, fish were stocked into twelve tanks (30 mixed-sex fish per tank) with a recirculating seawater system (2,025 liters, including biological filter). Six tanks were assigned randomly to normoxic conditions (dissolved oxygen, DO: 6.5 mg/L) and the other six tanks were maintained under hypoxic conditions (1.7 mg DO/L). The DO levels in the hypoxia exposure tanks were lowered by reducing the aeration gradually from 100–80% to 60, 40, and 20% through the air flow system. A YSI multiprobe meter (YSI 556 Multiprobe System, YSI Incorporated, Yellow Springs, OH) was used to monitor DO, pH, and temperature three times a day (morning, afternoon and night). A water quality kit (HACH, Loveland, CO) was used to measure ammonia and nitrite once a day. There were no major changes of water quality parameters (pH 7.7–7.9, ammonia 0.1–0.2 mg/L, and nitrite 0.01–0.02 mg/L) during the experimental periods. Fish in each tank were fed chopped shrimp (3% BW/day) and were sampled after continuous exposure for 1, 2 and 4 weeks to the target DO level (1.7 mg/L). At the end of experiments, fish were deeply anesthetized using quinaldine (20 mg/L) and the spinal cord severed following procedure approved by the University of Texas at Austin IACUC (protocol# 09022701). Liver tissues were rapidly excised, frozen in liquid nitrogen and stored at −80°C for later RNA extraction and protein determination. For immunohistochemical detection, liver samples were fixed in 4% paraformaldehyde (Sigma-Aldrich) overnight at 4°C.

### Experiment 2: Interactive Effects of Hypoxia and Exogenous NO-donor or NOS-inhibitor on Regulation of CYP1A

Thirty mixed-sex fish were stocked into each of four tanks and exposed to normoxic (6.5 mg DO/L) and hypoxic (1.7 mg DO/L) conditions. Fish were anesthetized with quinaldine as described above and injected i.p. either with vehicle, *S*-nitroso-N-acetyl-DL-penicillamine (SNAP, a NO-donor) or *N_ω_*-nitro-L-arginine methyl ester (NAME, a NOS-inhibitor) every 4 days for 4 weeks (6 injections of 1 µg SNAP or NAME/g BW). Approximately 20% of the water in each exposure tank was exchanged every week and there were no major changes of water quality parameters (same as in experiment 1) during the experimental period. Fish in each tank were fed the same amount of chopped shrimp (3% BW/day). All the food was consumed by the fish in the different treatment groups throughout the experiments. At the end of experiment, fish were anesthetized, and the liver tissues were excised and frozen in liquid nitrogen and stored at −80°C until analysis.

### Experiment 3: Interactive Effects of Hypoxia and an Antioxidant on Regulation of IL-1β, HIF-α, and CYP1A

Fish were stocked into each of four tanks (30 mixed-sex fish/tank) and exposed to normoxic (6.5 mg DO/L) and hypoxic (1.7 mg DO/L) conditions. Fish were anesthetized with quinaldine and injected i.p. either with vehicle or vitamin E, an antioxidant, every 4 days for 4 weeks (6 injections of 1 µg vit E/g BW). At the end of experiment, fish were anesthetized, and the liver tissues were frozen in liquid nitrogen and stored at −80°C for later analysis. All of these experiments were approved and conducted in accordance with animals use protocols by the University of Texas at Austin IACUC (protocol# 09022701).

### Cloning and Sequencing of CYP1A and IL-1β

Molecular protocols used for RNA extraction, cDNA synthesis, RT-PCR amplification, DNA purification, and cloning were similar to those of Rahman and Thomas [44]. Briefly, total RNA was extracted from croaker liver using TRI reagent and treated with DNase (Promega) to eliminate genomic DNA. First-strand cDNA synthesis was carried out using the RNA ligase mediated-RACE system (Invitrogen) according to the manufacturer’s protocol. Partial cDNA fragments of CYP1A and IL-1β were obtained by RT-PCR amplification. Degenerate primers (CYP1A primers: sense 5′-CCYMT CATTGGGAAYGTGCT-3′ and antisense 5′-GARTGGCGAAAGATYTCCAG-3′, and IL-1β primers: sense 5′-GRMATGCAACRTKAGCSAGA-3′ and antisense 5′-KSCTCT GMTGTGCTGATGTA-3′) for amplifying the cDNA fragments were designed to span highly conserved regions of the known sequences of CYP1A and IL-1β in teleost fishes and other vertebrates. The PCR products were separated by 1% agarose gel electrophoresis, purified and ligated into a pGEM-T easy vector (Promega), and then transformed into *E. coli* JM 109 competent cells (Promega). The plasmid DNA was purified using *Plus* SV Minipreps system (Promega) and sequenced (Institute for Cellular and Molecular Biology Core Research DNA Sequencing Facility, University of Texas at Austin). The full-length sequence of croaker CYP1A was obtained using 5′- and 3′-RACE amplifications system (Invitrogen) using gene-specific primers (5′-RACE primers: 5′-CTTGATGAGAGCCTGTCGAACTGT-3′ and nested 5′-CAACCTGGA TCTGGAAGACATCAC-3′, and 3′-RACE primers: 5′-TCTCTCTGACAGACCCAG CTTACC-3′ and nested 5′-AGCTTACCCTTCCTGGATGCTTTC-3′). The identity of croaker CYP1A was verified using the Basic Local Alignment Search Tool program and National Center for Biotechnology Information (NCBI, http://www.ncbi.nlm.nih.gov) database, and aligned using the ClustalW [45]. The multiple sequence alignments were adjusted manually to the regions corresponding to different substrate recognition and heme-binding sequences.

### Phylogenetic Analysis

A phylogenetic tree was constructed using the Neighbor-Joining approach according to Tamura et al. [46]. The consensus tree was developed by using the MEGA4 software package (http://megasoftware.net). Bootstrap analysis (1,000 replicates) was inferred to assess the degree of strength for branches on the tree. Only sequences that included a full coding region were used to construct the tree.

### Quantitative Real-time Polymerase Chain Reaction (qRT-PCR)

To determine the expression of croaker CYP1A, IL-1β and HIF-α mRNAs, qRT-PCR analyses were performed on total RNA using a Mastercycler quantitative real-time PCR system (Eppendrof, Westbury, NY). Gene-specific primers for croaker CYP1A (sense: 5′-TCAACGATGGCAAGAGTCTG-3′ and antisense: 5′-TACTCTGGGGTT GTGCCTTC-3′; GenBank accession number JQ622220), IL-1β (sense: 5′- CGTGACC GACAGTGAGAAGA -3′ and antisense: 5′- TCCCATCCTTATGGCAAGAG -3′ primers; JQ622219), HIF-2α (sense: 5′-ATCGCATGCTGGCTAAGAAC-3′ and antisense: 5′-CGAAGTCGAGGGAAATGATG-3′ primers; DQ363932) were used for amplification of croaker CYP1A, IL-1β and HIF-2α mRNA. The RNA samples were assayed using a one step of Brilliant SYBR Green qRT-PCR Master Mix Kit (Agilent Technologies, La Jolla, CA) in a 25-µl reaction mixture containing 12.5 µl 2x SYBR-qRT-PCR master mix, 50 nM of gene specific primers, 0.0625 µl StrataScript RT/RNase block enzyme mixture, and 250 ng of total RNA. The thermocycle profile was 50°C for 30 min, 95°C for 10 min, and 40 cycles of 95°C for 30 s, 55°C for 1 min, and 72°C for 30 s. Melting curve analysis was also included at one cycle of 95°C for 1 min, 50°C for 30 s, and 95°C for 30 s. Each transcript level was normalized on the basis of the quantification of croaker 18S rRNA (primers: sense 5′-AGAAACGGCTACCACATCCA -3′ and antisense 5′-TCCCGAGATCCAACTACGAG -3′; AY866435). To ensure that the qRT-PCR results were not the product of genomic DNA contamination, we also performed reverse transcriptase-negative qRT-PCR reaction. The resulting qRT-PCR data were converted into threshold cycle (C*t*) values, and the relative mRNA expression levels were analyzed using the 2^−ΔΔC*t*^ method according to Livak and Schmittgen [47].

### Preparation of Microsomes from Liver

Microsomal fractions were separated from liver tissue homogenates as described by Sullivan et al. [48]. Briefly, liver samples were homogenized in 1 ml of stabilization buffer (100 mM potassium phosphate containing 20% glycerol, 1 mM dithiothreitol, 1 mM EDTA; pH 7.4) containing 10 µl of halt protease inhibitor cocktail (Pierce, Rockford, IL) to minimize protein degradation. The homogenate was centrifuged at 12,000 *g* for 10 min. The pellets were then discarded and supernatants were further centrifuged at 100,000 *g* for 60 min using an ultracentrifuge (Beckman, Bera, CA) to yield microsomal pellets. The microsomal pellets were resuspended in stabilization buffer, immediately transferred into sterilized vials, and stored at −80°C until used for ethoxyresorufin-*O*-deethylase (EROD) assay and Western blot analysis.

### EROD Activity Assay

EROD activity was determined in the liver as an index of CYP1A enzymatic activity by measuring the microsomal conversion of ethoxyresorufin to resorufin according to Burke and Mayer [49] and Cavanagh et al. [50]. Briefly, the reaction mixtures were prepared daily containing 100 µl (0.01 mM) ethoxyresorufin substrate in a glass culture tube in ice bath, 250 µl of NADPH generating solution (10 mM MgCl_2_, 200 mM KCl, 5 mM glucose-6-phosphate, 1.25 mM NADP and 100U glucose-6-phosphate dehydrogenase), 525 µl Tris buffer (0.1 M Tris, pH 7.6) and 100 µl of 1.2 mg/ml albumin. The reaction mixture (975 µl) was vortexed and allowed to equilibrate in a water bath at 30°C for 2 min. Twenty five µl of microsomal preparation was then added to the reaction mixture, vortexed, and the mixture was incubated at 30°C for 10 min. The enzymatic reactions were terminated by adding 250 µl of ice-cold methanol and transfer to a ice bath followed by centrifugation at 2000 *g* for 5 min at 4°C. Resorufin concentrations were measured from 1 ml of blank, standard or assay supernatant using a Fluoro Star spectrophotometer (BMG Labtechnologies, Durham, NC) at 530/585 excitation/emission. Protein content in the assay supernatant was measured using the Bradford assay (Bio-Rad, Hercules, CA, USA) using BSA as the standard [51]. EROD activities were expressed as pmols min^−1^ mg protein^−1^.

### Western Blot Analysis

CYP1A and eNOS protein levels were determined in hepatic microsomal and cytosolic fractions, respectively, by Western blot analysis. Protein samples were solubilized by boiling in sodium dodecyl sulfate (SDS, Sigma-Aldrich) loading buffer (0.5 M Tris-HCl, 0.5% bromophenol blue, 10% glycerol) and cooled on ice for 5 min. Proteins were separated on a 10% SDS-polyacrylamide gel electrophoresis (SDS-PAGE) gel according to Laemmli [52], transferred onto a immuno-blot polyvinyl difluoride membrane (PVDF membrane, Bio-Rad) for at 4°C and blocked with 5% nonfat milk in Tris-buffer saline and Tween 20 (TBS-T: 50 mM Tris, 100 mM NaCl, 0.1% Tween 20, pH 7.4) for 2 h. Membranes were washed three times for 10 min with TBS-T buffer and probed with primary CYP1A and eNOS antibodies (dilution: 1∶1000) with 5% nonfat milk overnight at 4°C. The CYP1A antibody was generated against a highly conserved sequence of rainbow trout CYP1A, amino acids 277–294 [53], which is ∼89% identical to the corresponding region in Atlantic croaker (**supporting** **Fig. 2A**). The eNOS antibody has been validated in teleost ovaries previously [54]. Membranes were then washed with TBS-T, incubated for 2 h with anti-mouse IgG HRP-linked secondary antibody (1∶5,000; catalog no. 7076, Cell Signaling) for CYP1A and goat anti-rabbit IgG HRP-linked secondary antibody (1∶4,000; catalog no. 4050-01, Southern Biotech, Birmingham, AL) for eNOS proteins. Membranes were rinsed with TBS-T buffer, exposed to x-ray film by the addition of WestPico chemiluminescent substrate (Pierce, Rockford, IL) and photographed on Hyperfilm (Amersham Biosciences, Buckinghamshire, UK) in dark condition. The intensity of the CYP1A, eNOS and actin protein bands was estimated using ImageJ software (National Institutes of Health, Bethesda, MD; http://rsb.info.nih.gov/ij/). Actin protein (∼45 kDa) was used as an internal control to normalize sample loading on the gels.

### Immunohistochemistry

Liver samples were fixed in 4% paraformaldehyde overnight at 4°C, dehydrated in a series of increasing percent ethanol solutions, and embedded in paraffin (Paraplast, Sigma-Aldrich). Paraffin-embedded tissues were sectioned at 7 µm on a rotary microtome (Microm International GmbH, Walldorf, Germany). Sections were then deparaffinized with xylene, rehydrated with a series of decreasing percent ethanol solutions, and rinsed three times in phosphate-buffered saline (PBS). Endogenous peroxidase activity was blocked with 5% H_2_O_2_ for 10 min at room temperature. To expose the antigen, sections were boiled in a target retrieval solution (1 mM Tris, pH 9.0, with 0.5 mM EGTA) for 10 min. Nonspecific binding was prevented by blocking in PBS containing 1% BSA. The sections were rinsed three times in PBS and incubated with mouse monoclonal anti-CYP1A antibody (C10-7, [53]) at a dilution of 1∶100 overnight at 4°C. The fluorescence signal was amplified by adding Alexa Fluor 488 goat anti-mouse secondary antibody (dilution 1∶500, Invitrogen) for 1 h. The sections were rinsed three times in PBS, partially dried, and mounted with Prolong Gold antifade reagent (Invitrogen). The presence of the fluorescent-labeled CYP1A protein signal was examined using a Nikon Eclipse E600 microscope (Nikon, Japan) with fluorescein isothiocyanate (FITC; green) filter. The image was captured by Cool-SNAP camera (Photometrics, Tucson, AZ), and the immunostaining intensity of CYP1A expression was estimated using ImageJ software.

**Figure 1:**
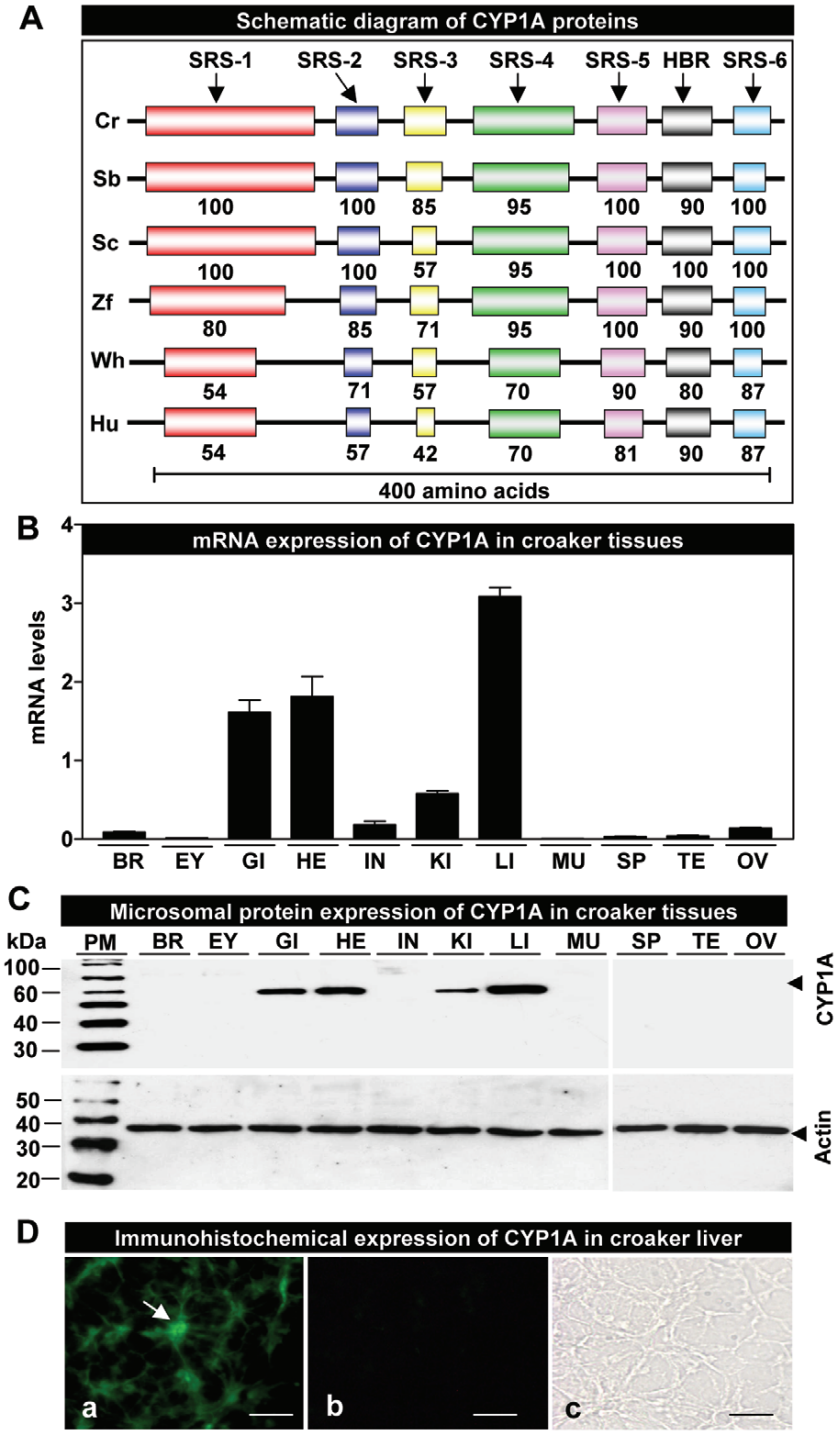
Structure, tissue distribution and immunohistochemical localization of croaker cytochrome P450 1A (CYP1A). (**A**) Schematic diagram of CYP1A proteins showing conserved substrate recognition sites (SRS 1–6) and heme-binding region (HBR). Numbers indicate percent identities of the predicted amino acid sequence of Atlantic croaker (Cr) CYP1A protein with those of CYP1As from other vertebrates. GenBank accession numbers for the sequences used are as follows: Cr, croaker (JQ622220); Sb, seabass (DLU78316); Sc, scup (U14162); Zf, zebrafish (AF210727); Wh, whale (AB231891); and Hu, human (K03191). (**B**) Expression of CYP1A mRNA in croaker tissues by qRT-PCR. Each value represents the mean±S.E.M (N = 4, tissues were randomly sampled from individual fish for measurements). (**C**) CYP1A protein expression in microsomes prepared from croaker tissues (Note: due to gel size limitations, equal amounts of microsomal protein from different tissues were run at room temperature under the same conditions on two separate gels). The positions of Western blot protein standard markers (PM) are indicated on the left. BR, brain; EY, eye; GI, gill; HE, heart; IN, intestine; KI, kidney; LI, liver; MU, muscle; OV, ovary; SP, spleen; TE, testis. D) Immunohistochemical CYP1A expression in croaker liver. Liver sections incubated with the primary and secondary antibodies showing the presence of immunoreactive signals (D-a, arrow indicates hepatocyte in liver tissue), and incubated with only secondary antibody (D-b) or only primary antibody (D-c) showing the absence of signals. Scale bar = 250 µm.

### Statistical Analysis

All of the experimental results were analyzed by one-way analysis of variance (ANOVA) and Fisher’s protected least-significant difference (PLSD) test for multiple comparisons and Student’s *t-*test for unpaired comparisons. Differences were considered significant if p<0.05. Analyses were performed using StatView (SAS Institute Inc., Cary, NC) and GraphPad Prism (GraphPad, San Diego, CA) software packages.

**Figure 2:**
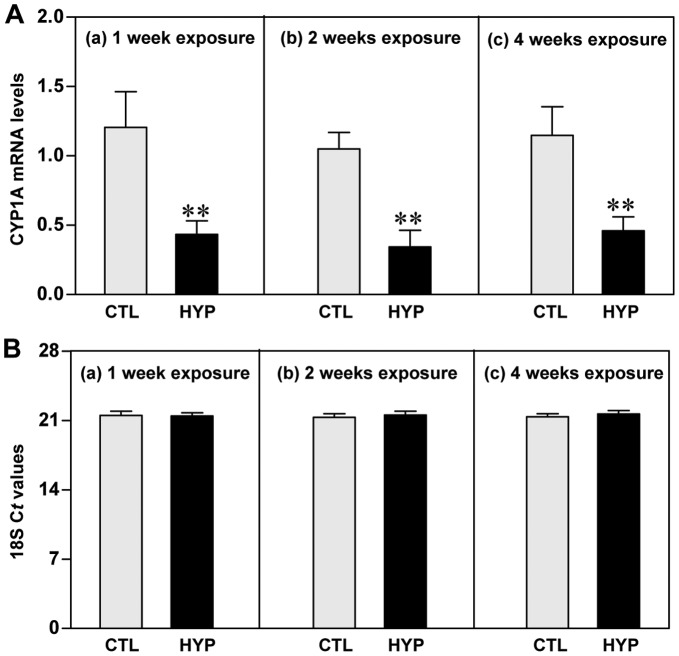
Hypoxia-induced expression of CYP1A mRNA determined by qRT-PCR. Effects of 1, 2 and 4 weeks hypoxia (dissolved oxygen, DO: 1.7 mg/L) exposure on CYP1A mRNA levels (**A**) and 18S rRNA expression (**B**) in croaker liver. Each value represents the mean±S.E.M (N = 8–10, tissues were randomly sampled from individual fish for measurements). Asterisk indicates significant difference from normoxic controls (Student’s *t*-test, **p<0.01). Note: exposure duration only refers to period fish were exposed to target DO; fish were previously exposed to declining DO for additional 2-day adjustment period. No significant difference was detected in 18S threshold cycle (C*t*) values between the treatment groups. CTL, control; HYP, hypoxia.

## Results

### Molecular Characterization of Croaker CYP1A

A distinct cDNA fragment of croaker CYP1A (998 bp) was identified by RT-PCR with degenerate primers. The complete cDNA sequence of croaker CYP1A was obtained by employing 5′- and 3′-RACE PCR. The full-length croaker CYP1A cDNA consists of 2581 bp nucleotides, which contains a 5′-untranslated region (5′-UTR, 174 bp), an open reading frame (1563 bp) encoding a polypeptide of 521 amino acid residues (**supporting** **Fig. 1**) with a predicted molecular weight of the protein of ∼59 kDa, and a 3′-UTR (844 bp). It has two pentanucleotide sequences, and a single poly-adenylated signal and poly(A) tail in the 3′-UTR. The nucleotide and amino acid sequences were deposited in GenBank (accession number JQ622220) and were then compared with those of other animals. The six substrate recognition sites (SRSs) and a heme-binding region (HBR) were determined in croaker CYP1A cDNA based of the SRSs and HBR data reported by Gotoh [55] and Poulos et al. [56], respectively. Like other teleosts, the croaker CYP1A protein shows extensive sequence homologies in the SRSs (71–100%) and HBR (90–100%) (**Fig. 1A**; **supporting** **Fig. 2A**). The deduced amino acid sequence of croaker CYP1A shows high identity with that of the seabass (89%), scup (85%) zebrafish (73%), whereas its sequence identity to whale and human is relatively low at 55 and 56%, respectively (**supporting** **Fig. 2B**).

**Figure 3:**
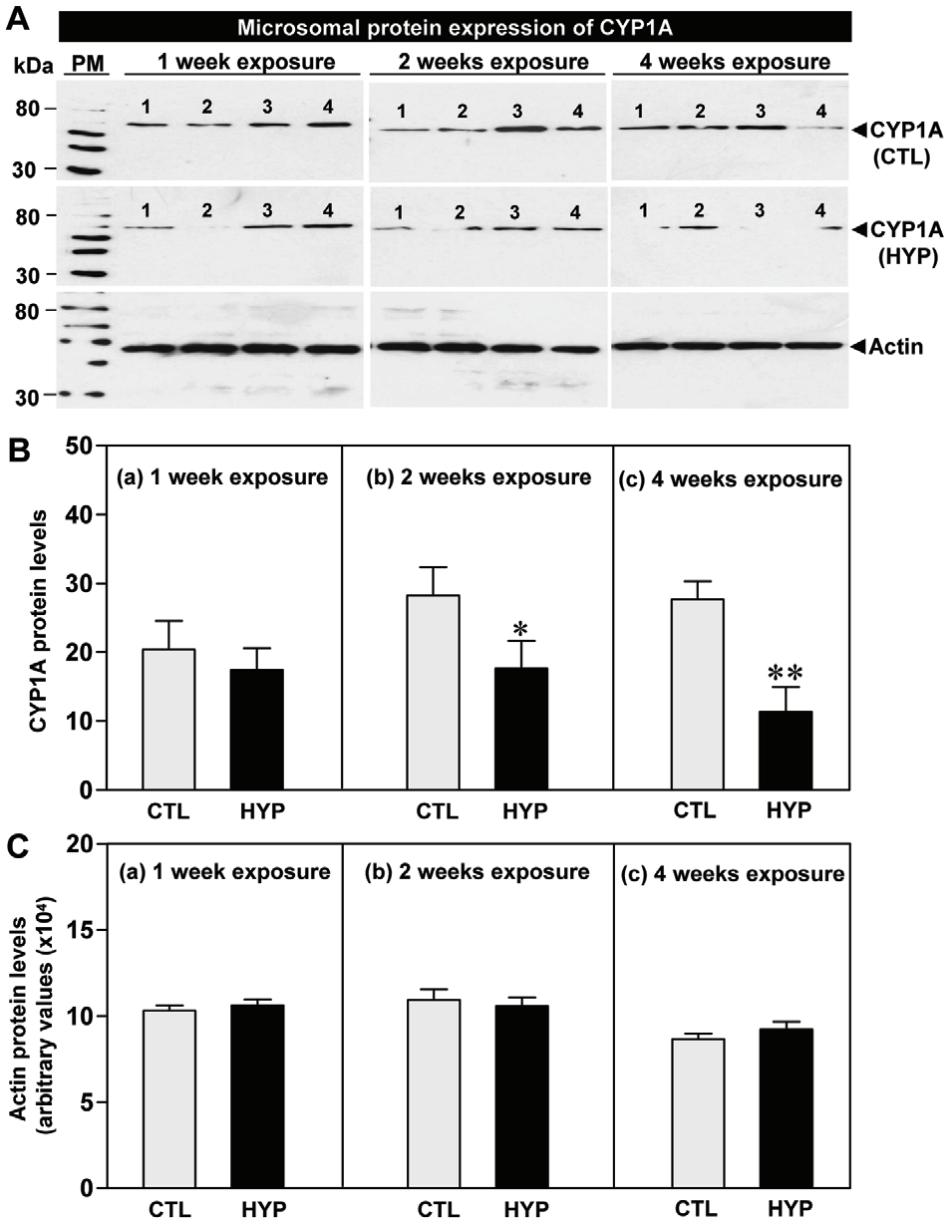
Hypoxia-induced expression of CYP1A protein determined by Western blot analysis. Effects of 1, 2 and 4 weeks hypoxia (dissolved oxygen, DO: 1.7 mg/L) exposure on CYP1A protein expression (**A**) and relative protein levels (**B**), and actin protein levels (**C**) in croaker liver. Each value represents the mean±S.E.M (N = 8–10, tissues were randomly sampled from individual fish for measurements). Asterisk indicates significant difference from normoxic controls (Student’s *t*-test, *p<0.05, **p<0.01). Note: exposure duration only refers to period fish were exposed to target DO; fish were previously exposed to declining DO for additional 2-day adjustment period. The positions of Western blot protein standard marker (PM) are indicated on the left. No significant difference was detected in actin protein levels between the treatment groups. CTL, control; HYP, hypoxia.

A phylogenetic tree of the deduced amino acid sequence was constructed to investigate the evolutionary relationship of croaker CYP1A to previously characterized vertebrate CYP1As. Thirty-seven teleost and six tetrapod species were evaluated in this study. CYP1A cDNAs from various species are clustered together as expected based upon other taxonomic studies (**supporting** **Fig. 3**). As expected the croaker CYP1A cDNA is more closely related to those of teleosts than those of tetrapods.

**Figure 4:**
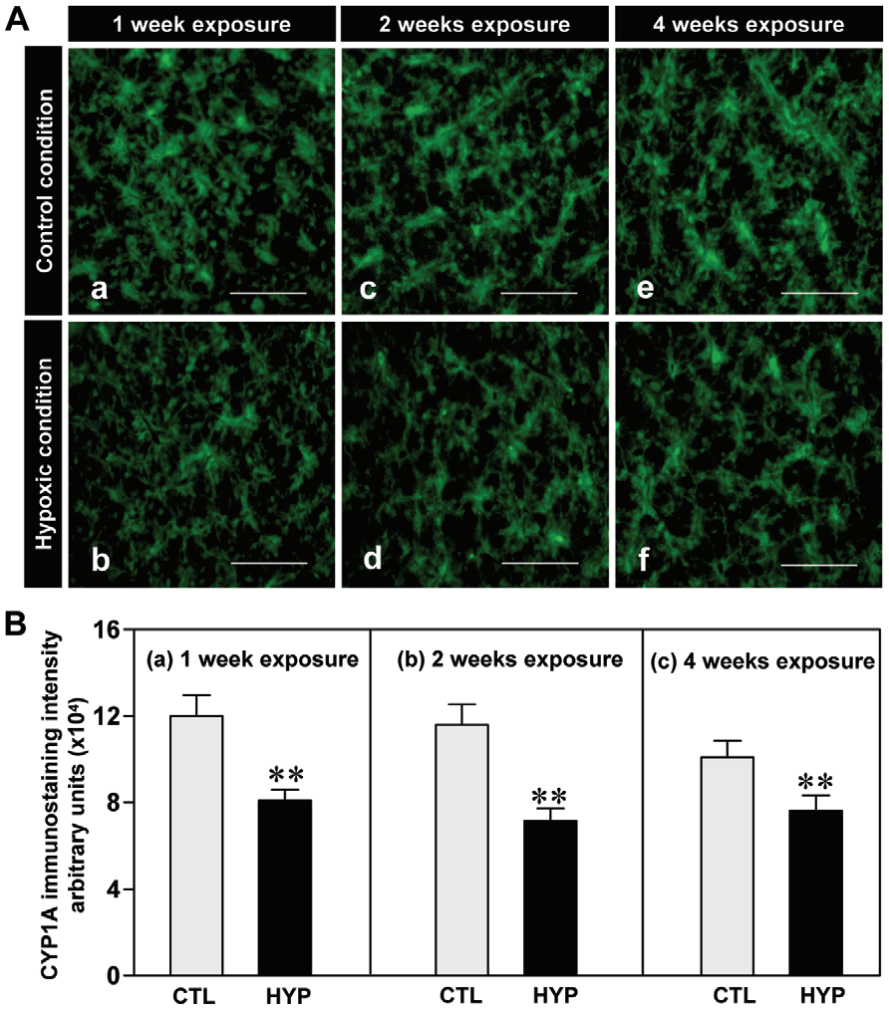
Hypoxia-induced expression of CYP1A protein assessed by immunohistochemistry. Effects of 1, 2 and 4 weeks hypoxia (dissolved oxygen, DO: 1.7 mg/L) exposure on immunohistochemical expression (**A**) and immunostaining intensity (**B**) of CYP1A in croaker liver. Each value represents the mean±S.E.M. Asterisk indicates significant difference from normoxic controls (Student’s *t*-test, **p<0.01). Note: exposure duration only refers to period fish were exposed to target DO; fish were previously exposed to declining DO for additional 2-day adjustment period. CTL, control; HYP, hypoxia. Scale bar = 250 µm.

### Tissue-specific Expression of CYP1A mRNA and Protein

Quantitative real-time PCR (qRT-PCR) results of tissues collected from croaker exposed to normoxic conditions revealed that CYP1A mRNA is highly expressed in the liver, heart and gill, whereas expression is lower in the kidney and intestine and much lower in the brain, spleen, testis, and ovary. CYP1A mRNA was not detected in the eye and muscle (**Fig. 1B**).

**Figure 5:**
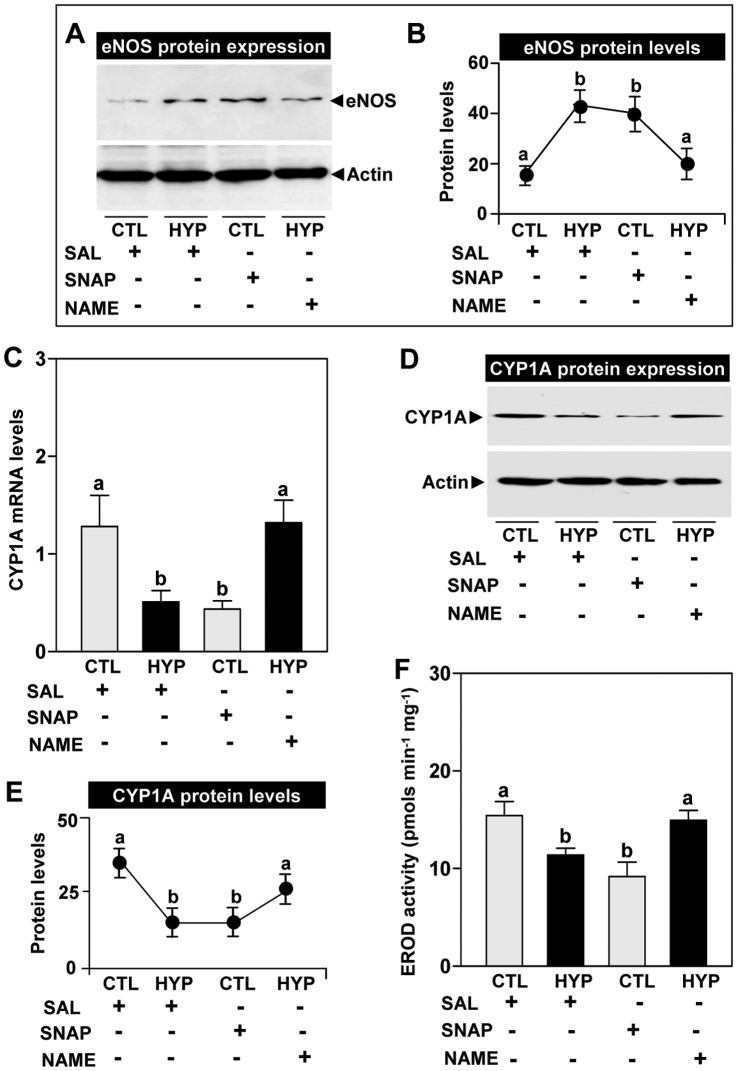
Interactive effects of hypoxia and NO drugs on eNOS and CYP1A expression. Effects of 4 weeks hypoxia (dissolved oxygen, DO: 1.7 mg/L) exposure and pharmacological treatments with *S*-nitroso-N-acetyl-DL-penicillamine (SNAP, a donor of nitric oxide) and *N_ω_*-nitro-L-arginine methyl ester (NAME, an inhibitor of nitric oxide synthase) on endothelial nitric oxide synthase (eNOS) protein expression (**A**) and eNOS protein levels (**B**), CYP1A mRNA levels (**C**), CYP1A protein expression (**D**), CYP1A protein levels (**E**), and ethoxyresorufin *O*-dethylase (EROD) activity (**F**) in croaker livers. Each value represents the mean±S.E.M (N = 8–9, tissues were randomly sampled from individual fish for measurements). Significant differences identified with a multiple range test, Fisher’s PLSD, are indicated with different letters (p<0.05). Note: exposure duration only refers to period fish were exposed to target DO; fish were previously exposed to declining DO for additional 2-day adjustment period. CTL, control; HYP, hypoxia; SAL, saline.

Western blot analysis using the CYP1A monoclonal antibody showed a single immunoreactive band of the predicted size ∼59 kDa in croaker tissue extracts (**Fig. 1C**). The CYP1A protein was highly expressed in the liver, heart and gill microsomal fractions and a weak band was apparent in kidney preparations. No immunoreactive bands were detected in other tissue preparations. Equal loading of the microsomal fractions for different tissues was confirmed with the actin loading control (**Fig. 1C**).

**Figure 6:**
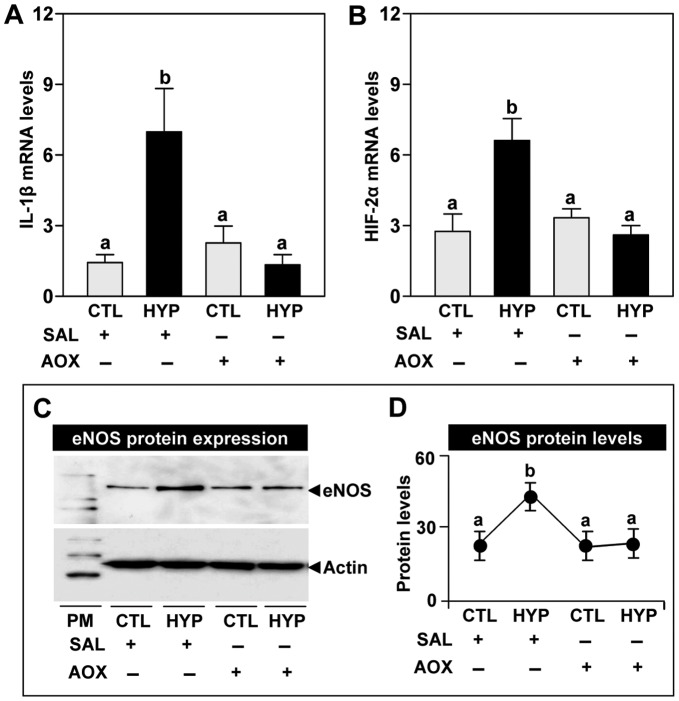
Interactive effects of hypoxia and an antioxidant on IL-1β, HIF-2α and eNOS expression. Effects of 4 weeks hypoxia (dissolved oxygen, DO: 1.7 mg/L) exposure and antioxidant (AOX, vitamin E) treatment on interleukin-1β (IL-1β) mRNA levels (**A**), hypoxia-inducible factor-2α (HIF-2α) mRNA levels (**B**), endothelial nitric oxide synthase (eNOS) protein expression (**C**), and eNOS protein levels (**D**). Each value represents the mean±S.E.M (N = 8–10, tissues were randomly sampled from individual fish for measurements). Significant differences identified with a multiple range test, Fisher’s PLSD, are indicated with different letters (p<0.05). Note: exposure duration only refers to period fish were exposed to target DO; fish were previously exposed to declining DO for additional 2-day adjustment period. CTL, control; HYP, hypoxia; SAL, saline.

Because CYP1A is known to play important physiological roles in the liver, we further examined the expression pattern of the CYP1A protein in croaker liver by immunohistochemistry. Positive fluorescence signals were observed in hepatocytes with the CYP1A antiserum (**Fig. 1D** **-a**). No signal was detected in control sections when the primary or secondary antibodies were omitted (**Fig. 1D-b, 2D-c**).

### Effects of Short- and Long-term Hypoxia Exposure on CYP1A mRNA and Protein Levels

qRT-PCR results showed that CYP1A mRNA levels were significantly decreased after short- (1 week) and long-term (2 and 4 weeks) hypoxia exposure (1.7 mg DO/L) compared to normoxic controls (**Fig. 2A**). The 18S C*t* values in croaker livers were the same in the normoxic control and hypoxia exposure groups (**Fig. 2B**).

**Figure 7:**
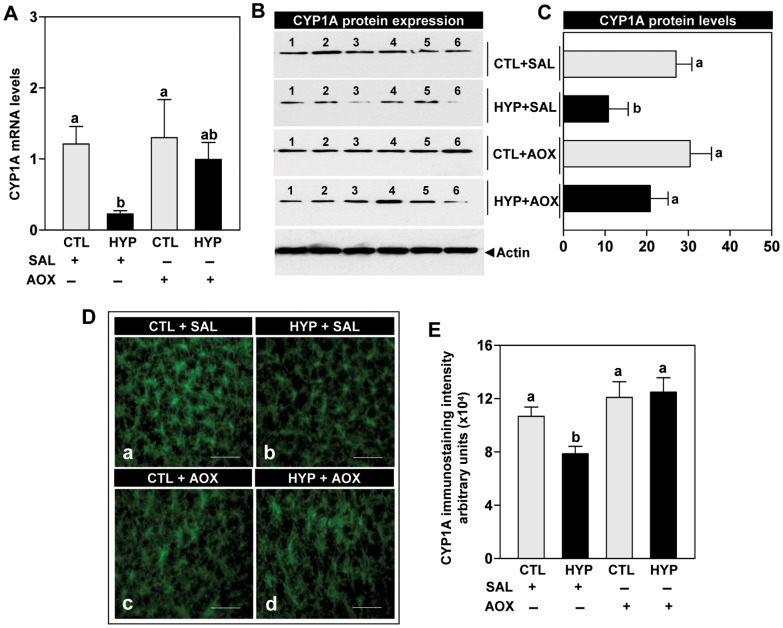
Interactive effects of hypoxia and an antioxidant on CYP1A expression. Effects of 4 weeks hypoxia (dissolved oxygen, DO: 1.7 mg/L) exposure and antioxidant (AOX, vitamin E) treatment on CYP1A mRNA levels (**A**), CYP1A protein expression (**B**; Note: representative Western blot shown for samples from individual fish), CYP1A protein levels (**C**), immunohistochemical expression (**D**) and immunostaining intensity (**E**) of CYP1A in croaker liver. Each value represents the mean±S.E.M (N = 8–12, tissues were randomly sampled from individual fish for measurements). Significant differences identified with a multiple range test, Fisher’s PLSD, are indicated with different letters (p<0.05). Note: exposure duration only refers to period fish were exposed to target DO; fish were previously exposed to declining DO for additional 2-day adjustment period. CTL, control; HYP, hypoxia; SAL, saline. Scale bar = 400 µm.

CYP1A protein levels in liver microsomal fractions were not significantly altered after short-term hypoxia exposure (**Fig. 3A, 3B** **-a**); whereas, longer-term exposure to hypoxia for two and four weeks caused significant decreases in CYP1A protein levels in liver compared to the normoxic controls (**Fig. 3A, 3B** **-b, 3B-c**). Actin protein levels (loading controls) were the same in liver tissues from control and hypoxia-exposed fish (**Fig. 3C**).

**Figure 8:**
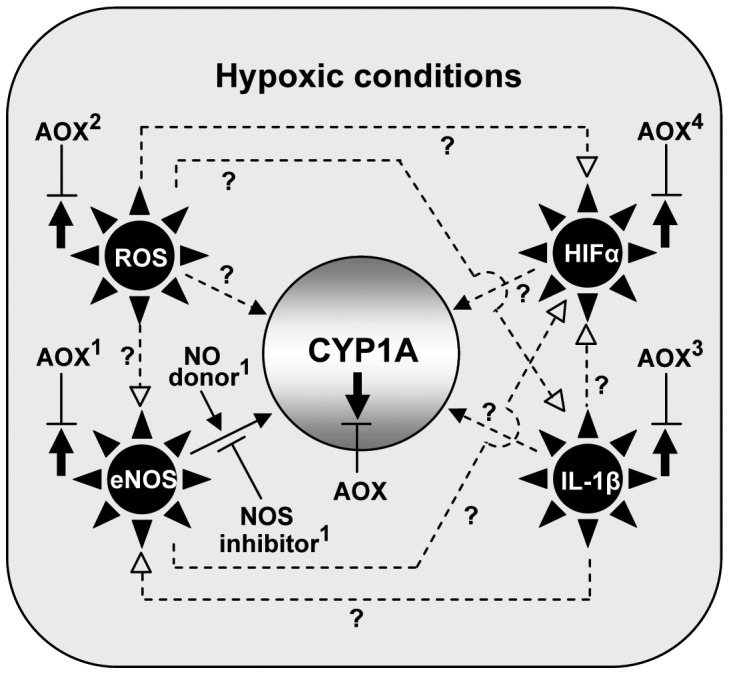
Proposed mechanisms of hypoxia down-regulation of CYP1A expression. Summary of results of the current study (shown as solid lines) and proposed model of hypoxia down-regulation of croaker hepatic CYP1A expression (shown as dotted lines). The results show antioxidant (AOX) treatment blocks the hypoxia-induced down-regulation of CYP1A expression (solid arrow pointing down) and upregulation of endothelial nitric oxide synthase (eNOS), hypoxia-inducible factor-α (HIFα), and interleukin-1β (IL-1β) (^1,3,4^present study, shown as solid arrows pointing up). In addition treatment with a NO-donor (SNAP) mimics the effects of hypoxia on CYP1A expression, whereas treatment with a NOS-inhibitor (NAME) reverses the inhibitory effects of hypoxia, suggesting an involvement of eNOS in CYP1A regulation. Antioxidant treatment also blocks the hypoxia-induced increase in reactive oxygen species (ROS) production (^2^Rahman and Thomas [43]). The model shows several pathways (dotted lines) through which hypoxia could potentially down-regulate CYP1A expression in croaker. ‘?’: evidence has only been obtained in mammalian *in vitro* studies.

Immunohistochemical detection of the CYP1A protein in croaker liver sections showed that immunoreactive CYP1A expression appeared to be weaker in both the short- and long-term hypoxia treatment groups compared to normoxic controls (**Fig. 4Aa–f**). The intensity of immunostaining of CYP1A protein decreased ∼25–38% in liver tissues of hypoxia-exposed fish compared to normoxic controls (**Fig. 4Ba–c**).

### Potential Mechanisms of CYP1A Down-regulation

#### Pharmacological treatments with a NO-donor and NOS-inhibitor on CYP1A expression and EROD activity

Exposure to hypoxia (1.7 mg DO/L) and the NO-donor, SNAP for four weeks, caused similar increases in hepatic eNOS protein expression compared to normoxic and saline controls, respectively, whereas the NOS-inhibitor, NAME, attenuated the hypoxia-induced increase in eNOS protein levels (**Fig. 5A, 5B**). qRT-PCR results showed that the NO-donor caused a similar decrease in CYP1A mRNA levels as that observed after hypoxia exposure and that the hypoxia effect was reversed by treatment with the NOS-inhibitor (**Fig. 5C**). Similarly, exposure to hypoxia and the NO-donor caused comparable decreases in microsomal CYP1A protein expression (**Fig. 5D**) and protein levels (**Fig. 5E**) compared to normoxic and saline controls, respectively, whereas the NOS-inhibitor reversed the hypoxia-induced down-regulation of CYP1A protein expression and levels (**Fig. 5D, 5E**). A similar pattern of treatment effects was observed for in hepatic EROD activity after exposure to hypoxia and the NO drugs (**Fig. 5F**).

#### Pharmacological treatments with an antioxidant on hypoxia regulation of IL-1β, HIF-α, and CYP1A

In order to investigate the interactive effects of an antioxidant, vitamin E, with hypoxia on the regulation of IL-1β mRNA, we partially cloned croaker IL-1β cDNA (**supporting** **Fig. 4**, GenBank accession number JQ622219). Exposure to hypoxia caused a significant increase in IL-1β mRNA levels in croaker livers (**Fig. 6A**). Injection with vitamin E had no effect on IL-1β mRNA levels in normoxic fish, but the hypoxia-induced increase in IL-1β mRNA levels was completely blocked by the antioxidant treatment (**Fig. 6A**). HIF-2α mRNA levels in croaker livers were increased around 2-fold in hypoxia-exposed fish compared to normoxic controls, whereas treatment with vitamin E blocked the hypoxia-induced increase in HIF-2α mRNA levels (**Fig. 6B**). Similarly, treatment with vitamin E blocked the hypoxia-induced increase in hepatic eNOS protein expression and levels (**Fig. 6C, 6D**).

qRT-PCR results showed that CYP1A mRNA levels were partially reversed by treatment with vitamin E in hypoxia-exposed fish, restoring them to near normoxic control levels (**Fig. 7A**). Western immunoblot results showed that CYP1A protein expression and levels were restored in the livers of hypoxia-exposed fish to normoxic control levels by treatment with vitamin E (**Fig. 7B, 7C**). Similarly, immunoreactive CYP1A protein expression and staining intensity were restored in hepatic tissues of hypoxia-exposed fish by treatment with vitamin E (**Fig. 7D, 7E**).

## Discussion

In this report we describe the molecular characterization of CYP1A from a marine teleost, Atlantic croaker, and potential mechanisms of hypoxia down-regulation of CYP1A expression. Chronic exposure (4 weeks) to hypoxia (1.7 mg DO/L) caused significant declines in CYP1A mRNA and protein expression and also increased IL-1β and HIF-2α mRNA expression and eNOS protein levels in croaker livers. The finding that injections of vitamin E (1 µg vit E/g BW/4 days) attenuated the hypoxia-induced increases in eNOS, IL-1β, HIF-2α and reversed the hypoxia down-regulation of CYP1A expression suggests these hypoxia responses are mediated by alterations in hepatic antioxidant status. Vitamin E treatment has previously been shown to reverse the hypoxia-induced increase in ROS production in croaker livers [43]. Taken together the results obtained with vitamin E implicate ROS, IL-1β, HIF-α and eNOS as potential intermediaries in hypoxia down-regulation of CYP1A whose actions are blocked by antioxidants. These proposed mechanisms of CYP1A down-regulation and their inhibition by antioxidant treatment are summarized in Figure 8. The experiments with SNAP (NO-donor) and NAME (NOS-inhibitor) provide direct evidence for an involvement of eNOS and NO in CYP1A regulation under hypoxic and normoxic conditions. To the best of our knowledge, this is the first evidence for a role of NO and antioxidants in hepatic CYP1A regulation in any aquatic vertebrate during hypoxic stress.

The CYP1A cDNA cloned and sequenced from croaker liver in the present study has a long untranslated region (844 bp nucleotides) at the 3′ prime end similar to that reported in other teleost species [57]–[60]. Analysis of the nucleotide and deduced amino acid sequences of croaker CYP1A (**supporting** **Fig. 1** **,** **2**) indicates several regions characteristic of teleost CYP1As including the proline-rich region downstream of the amino terminal, which is thought to be crucial for the conformation of the CYP1A isoform [61], and a well conserved HBR domain (80–100%), which operates as the fifth ligand binding region of heme in CYP1A protein [55]. The high degree of sequence conservation in the SRS regions between croaker and other teleosts suggests the substrate specificity of croaker CYP1A is similar to that of other teleost species [55]. As expected, phylogenetic analysis shows that the croaker CYP1A isoform is clustered with those of other perciform fishes (**supporting** **Fig. 3**), which comprise one of the largest teleost orders.

The expression patterns of CYP1A mRNA and protein observed in the tissues of croaker maintained under normoxic conditions in the laboratory for several months and not treated with xenobiotic chemicals are consistent with the known functions of the enzyme. Highest expression of both CYP1A mRNA and protein was observed in croaker livers, the major site of biotransformation of xenobiotics and endogenous compounds [62], where high expression has been reported in other teleost fishes [4], [62]. Similar to findings in other teleosts, the gills, which are important organs for assimilation of oxygen and xenobiotics, and the heart and kidney also had relatively high CYP1A expression [63], [64]. Expression of CYP1A was low or undetectable in other tissues.

An important finding of this study is that chronic exposure (1–4 weeks) to hypoxia (23% O_2_-saturation) causes marked declines in hepatic CYP1A mRNA and protein expression in croaker which were accompanied by a significant decrease in EROD activity. Similarly, long-term (6 weeks) hypoxia exposure (46.2% O_2_-saturation) caused a reduction in the expression of hepatic CYP1A mRNA in Atlantic cod [27]. In addition, shorter-term hypoxia exposure (21% O_2_-saturation) for three days has been shown to decrease EROD activity in zebrafish larvae [26]. In contrast, hepatic CYP1A mRNA levels were not significantly altered in juvenile orange-spotted grouper after both short-term (3, 7 days) and long-term (2–4 weeks) hypoxia (20–30% O_2_-saturation) exposure [65]. Hepatic expression of CYP1A and other CYPs and hepatic CYP activity are also down-regulated after hypoxia exposure in mammals [28], [66]. It is concluded from these studies that the reduction in hepatic CYP1A expression and EROD activity is a common phenomenon but not an ubiquitous consequence of hypoxia exposure in teleost fishes.

One of the most interesting discoveries in the present study is the evidence for an involvement of eNOS and NO in the mechanism of hepatic CYP1A down-regulation by hypoxia. In a series of *in vivo* studies, exposure to hypoxia and administration of SNAP, a NO-donor, were shown to cause similar decreases in hepatic EROD activity, CYP1A mRNA and protein levels in croaker compared to saline control normoxic-fish. We have recently observed that hypoxia leads to increases in the concentration of NO metabolites (NOx, nitrite and nitrate) in croaker plasma (Rahman and Thomas, unpublished results), which is consistent with the results of previous studies showing that hypoxia increases NOx levels in rainbow trout plasma [67] and in rat livers [39]. Moreover, several mammalian *in vitro* studies have been shown that treatment with various NO donors such as SNAP, *S*-nitroprusside, and *S*-nitrosoglutathione, leads to suppression of CYP1A1 and CYP1A2 activities and their mRNA and protein expression in rat hepatocytes under nonhypoxic conditions [31], [68]. For example, NO enhanced the degradation of hepatic CYP1A1 and CYP1A2 proteins, and suppressed their mRNA expression and enzymatic activities in rats, while treatments with a NOS-inhibitor, NAME or aminoguanidine, restored CYP1A1 and CYP1A2 activities along with their protein levels [30], [69]. In the present study, administration of NAME completely reversed the down-regulation of hepatic CYP1A activity, mRNA and protein levels in hypoxia-exposed fish. These results provide direct evidence for a NO-dependent mechanism for down-regulation of CYP1A expression in croaker after hypoxia exposure. In addition, treatment with an antioxidant was shown to decrease eNOS expression and restore CYP1A levels in croaker livers. Taken together, our findings and the results in mammals suggest that hypoxia causes elevated NO and nitrogen radical levels through an increase in NO synthase (e.g. eNOS) activity resulting in increased oxidative stress, which in turns leads to decreased hepatic CYP1A expression; hypoxic actions blocked by vitamin E treatment (mechanism 1, **Fig. 8**).

The superoxide radical (SOR, O_2_•^−^, an index of ROS) directly suppresses CYP activity in rabbit hepatocytes *in vitro* and increased SOR production leads to reductions in CYP1A1 activity in rat liver *in vivo* [70], [71]. In addition, Barker et al. [72] demonstrated that exogenous hydrogen peroxide (H_2_O_2_, an index of ROS generation) decreases CYP1A1 and CYP1A2 mRNA levels in cultured rat hepatocytes. Interestingly, SOR can directly interact with NO to generate peroxynitrite, a reactive nitrogen species (RNS) which inactivates CYP proteins by nitrotyrosilation [73], [74]. Treatment with 21-aminosteroid, a potent inhibitor of membrane lipid peroxidation and a free radical scavenger [75], prevents the hypoxia-induced down-regulation of hepatic CYP activity in rabbits [66]. Hypoxia exposure also leads to increased hepatic ROS production *in vivo* in croaker which is blocked by treatment with vitamin E [43]. Therefore, a second potential mechanism of hypoxia-induced suppression of hepatic CYP1A is through increased production of ROS and elevated oxygen radical levels, a mechanism that can be reversed by antioxidant treatment (mechanism 2, **Fig. 8**).

Arguably, the most significant finding of this study is that hypoxia causes marked increases in the hepatic mRNA levels of IL-1β, a cytokine and key mediator of inflammation. The fact that this increase in IL-1β mRNA is accompanied by increases of hepatic HIF-2α mRNA and eNOS protein levels and a decrease in CYP1A levels is consistent with the proposed role of IL-1β in their regulation during hypoxia stress [28], [30], [76]. In addition to hypoxia-derived cellular reactive free radicals such as ROS and RNS, cellular CTK also promote oxidative modifications and play an important role in hepatic cell injury as well as inhibition of hepatic CYPs activity [28]. It has been shown that CTK such as IL-1β, tumor necrosis factor, and interferon-γ, are synchronously increased in mammals during hypoxia and that elevated CTK transiently enhance the production of SOR via activation of NADPH oxidase [76], and that this oxidative burst involves a rapid decrease of hepatic CYPs activity [28]. Importantly, the present results show that the upregulation of croaker hepatic IL-1β mRNA is prevented by treatments with vitamin E, a potent antioxidant. These findings are consistent with studies in mammals showing that these cytokine-induced chain reactions can be terminated using antioxidants such as vitamin A or vitamin E, by removing free radical intermediates [77], [78]. Antioxidants are prototypical scavengers of ROS, and attenuate CTK-induced HIF-α activation, block NADPH-oxidase by scavenging SOR, and reduce HIF-α activity [76]. This is in agreement with our findings which suggest that during hypoxia stress, vitamin E prevents the upregulation of IL-1β, HIF-α transcription and eNOS translation, and leads to decreased cellular NO and SOR generation, resulting in the restoration of hepatic CYP1A levels. Therefore, IL-1β is proposed as another mediator of hypoxia-induced down-regulation of CYP1A in croaker livers through its upregulation of HIF-α and NOS, CTK actions that can be blocked by antioxidants (mechanisms 3, 4; **Fig. 8**).

### Conclusion

This study provides the first clear evidence that hepatic NO and antioxidant status play important roles in the regulation of hepatic CYP1A expression during hypoxic stress in teleosts. The finding that the increases in eNOS, IL-1β, ROS and HIF-α during hypoxia are reversed by antioxidant treatment suggests they are potential intermediaries in hypoxia-induced down-regulation of CYP1A as shown in the proposed model. However, it is not known whether increases in IL-1β and HIF-α expression, and NO and ROS generation act in concert to inhibit hepatic CYP1A expression during hypoxic stress. Additional pharmacological approaches and time-course studies will be required to determine the relative importance of each of these proposed mechanisms and the likely sequence of events. Finally, Atlantic croaker is frequently used as an indicator species for environmental contamination of estuaries because it inhabits harbors and ship channels where organic xenobiotic chemical contamination is greatest. However, the bottom waters of these estuarine regions where this bentho-pelagic species is found are often seasonally hypoxic. Therefore, the present findings potentially have important implications for the use of CYP1A as an indicator of environmental contamination in croaker and other estuarine teleost species during seasonal hypoxic events and also for the toxicological impacts of xenobiotics on these organisms during this period when xenobiotic biotransformation may be compromised.

## Supporting Information

Figure S1 **Nucleotide and deduced amino acid (below the former) sequences of Atlantic croaker cytochrome P450 1A gene.** The start codon, heme-binding cysteine codon (position 463), stop codon (TAA), ATTTA (AUUUA) pentanucleotide sequences, TATA box, and putative poly-adenylation signal (AAAATAAAA) are all underlined and boldfaced. GenBank accession number JQ622220.
(PDF)

Figure S2 **Amino acid sequence of CYP1A protein.** Alignment of the full-length amino acid sequence of Atlantic croaker cytochrome P450 1A (CYP1A) with CYP1A-related protein of other vertebrates (A). Amino acid sequence identity (%) between croaker CYP1A and those of other vertebrates (B). Dots indicate residues that are identical to croaker CYP1A1. Dashes indicate gaps introduced to facilitate alignment. GenBank accession numbers for the sequences used are as follows: croaker (JQ622220), seabass (DLU78316), scup (U14162), rainbow trout (T.) (M21310), rainbow trout (M21310), zebrafish (AF210727), whale (AB231891) and human (K03191). The substrate recognition site (SRS) and heme-binding region (HBR) are underlines.
(PDF)

Figure S3 **Molecular phylogeny of CYP1A protein.** The phylogenetic tree was constructed using the Neighbour-Joining method (1000 bootstrap). Bootstrap values are shown at the branch points. Scale bar indicates the number of changes inferred as having occurred along each branch. GenBank accession numbers for European seabass (CAB63650), Japanese seabass (ADC35580), tiger bass (ABZ88704), Atlantic croaker (JQ622220), large yellow croaker (ACT64126), four-eye butter flyfish (Q92039), scup (U14162), gilthead seabream (O42457), sand steenbras (AAK69390), black porgy (ABI54450), Japanese flounder (ABO38813), marbled flounder (BAC87834), winter flounder (ADV36120), common dab (O42430), European flounder (Q9YH64), European plaice (Q92100), killifish (AAD01809), mangrove rivulus (AAQ16634), Japanese medaka (AAP48792), flathead mullet (ABZ88706), golden gray mullet (O42231), thicklip gray mullet (ABD95933), leaping mullet (Q9W683), pufferfish (ABV24057), spotted green pufferfish (CAG03127), three spined stickleback (ADO15701), spotted snakehead (ACL31529), Atlantic salmon (AAM00254), brook trout (AAQ10899), lake trout (AAQ10900), rainbow trout (AAD14035), Japanese eel (BAA88241), European eel (AAL99904), yellow catfish (EF584508), rare minnow (ABV01348), goldfish (ABF60890), zebrafish (NP_571954), African clawed frog (BAA37079), tropical clawed frog (AAI35261), guinea pig (NP_001166411), mouse (Y00071), whale (AB231891) and human (K03191).
(PDF)

Figure S4 **Nucleotide and deduced amino acid (below the former) sequences of Atlantic croaker interleukin-1β cDNA.** Putative *N*-glycosylation site is underlined. GenBank accession number JQ622219.
(PDF)
