# Supplementary material for: Effects of Hypoxia Exposure on Hepatic Cytochrome P450 1A (CYP1A) Expression in Atlantic Croaker: Molecular Mechanisms of CYP1A Down-Regulation
Source: PLoS One. 2012 Jul 16;7(7):e40825. doi: 10.1371/journal.pone.0040825 (PMC3397942; doi:10.1371/journal.pone.0040825)
Supplement: Figure S1 — Nucleotide and deduced amino acid (below the former) sequences of Atlantic croaker cytochrome P450 1A gene. The start codon, heme-binding cysteine codon (position 463), stop codon (TAA), ATTTA (AUUUA) pentanucleotide sequences, TATA box, and putative poly-adenylation signal (AAAATAAAA) are all underlined and boldfaced. GenBank accession number JQ622220. (PDF) [file pone.0040825.s001.pdf]

TCAAGAGAGTAAACTCGGACAATAACTTTCCACTTCCACAACGATATTTTCTTTACTGTTACTATTTGCTTTCCTGTGTTTACTAC 87
AAAGTGCCTGTGGAACCGCAACATTGTTCCTATTCAACCACTCGACTAAGGTTGCGCCGGAGCCAGAAAAAAAAAGGAGTTGTCATC 174
ATGGTGCTAATGATACTGCCATTCATTGGATCAGTGTCGGTATCTGAGAGTTTGGTGGCC 261
M V L M I L P F I G S V S V S E S L V A 20
ATAACAGTGTTGTGTGTGGTCTACCTGGCTCTCAGGTGTTTTCACACTGAAATTCCTGAGGGGCTTCGTCGACTGCCTGGACCAAAG 348
I T V L C V V Y L A L R C F H T E I P E G L R R L P G P K 49
CCCTTTCCTCTCATTGGGAATGTGCTGGAAGTGGGCAGCAAACCCTACTTGAGTCTCACTGCCATGAGCAAGCGTTATGGTGATGTC 435
P F P L I G N V L E V G S K P Y L S L T A M S K R Y G D V 78
TTCCAGATCCAGGTTGGCATGCGTCCGGTCATTGTGTTGAGTGGCACTGACACAGTTCGACAGGCTCTCATCAAGCAAGGGGAAGAG 522
F Q I Q V G M R P V I V L S G T D T V R Q A L I K Q G E E 107
TTTGCAGGCAGACCTGACCTGTACAGCTTCAGGTTCATCAACGATGGCAAGAGTCTGGCCTTCAGTACAGACCAGGCCGGCGTGTGG 609
F A G R P D L Y S F R F I N D G K S L A F S T D Q A G V W 136
CGTGCTCGCAGGAAGCTGGCCTACAGTGCCCTGCGCTCCTTCTCCAGCCTGGAAGGCACAACCCCAGAGTACTCCTGCATGCTGGAA 696
R A R R K L A Y S A L R S F S S L E G T T P E Y S C M L E 165
GAACACATTTGCAAAGAGGGAGAATATCTGATCAAACAGCTCAACAATGTCATGAAGGCTGACGGCAGCTTCGACCCCTTCCGACAC 783
E H I C K E G E Y L I K Q L N N V M K A D G S F D P F R H 194
ATTGTTGTCTCCGTTGCCAATGTGATCTGTGGAATGTGCTTCGGCCGACGCTATGACCACAATGACCAGGAGCTTCTCAGCTTGGTG 870
I V V S V A N V I C G M C F G R R Y D H N D Q E L L S L V 223
AACCTCAGTGACGAATTCGGTCAGGTGGTGGGCAGCGGTAACCCTGCAGACTTCATCCCTATTCTCCAATACCTGCCCAGCTCAACA 957
N L S D E F G Q V V G S G N P A D F I P I L Q Y L P S S T 252
ATGAAGACTTTCATGGACATCAATGTCCGCTTCAACACATTTGTGCAAAAGATCGTCAGTGAGCACTATGCCAACTATAACAAGGAC 1044
M K T F M D I N V R F N T F V Q K I V S E H Y A N Y N K D 281
AACATTCGTGACATCACTGATTCCCTCATTGATCACTGTGAGGACAGAAAGCTGGATGAGAACTCCAATGTCCAGATGTCAGATGAG 1131
N I R D I T D S L I D H C E D R K L D E N S N V Q M S D E 310
AAGGTTGTAGGCATCGTCAATGACCTGTTCGGAGCTGGTTTTGACACCATCTCCACTGCCCTGTCTTGGTCAGTAATGTACTTGGTG 1218
K V V G I V N D L F G A G F D T I S T A L S W S V M Y L V 339
GCTCACCCAGAGATGCAGGAAAGGCTTTATCAAGAACTGAAGGACACAGTGGGTCTGGATCGCAGTCCTCGTCTCTCTGACAGACCC 1305
A H P E M Q E R L Y Q E L K D T V G L D R S P R L S D R P 368
AGCTTACCCTTCCTGGATGCTTTCATCCTGGAGATCTTTCGCCACTCTTCATTCCTGCCCTTCACCATCCCTCACTGCACATCAAAA 1392
S L P F L D A F I L E I F R H S S F L P F T I P H C T S K 397
GACACATCGCTAAATGGATACTTCATTCCCAAAGACACCTGTGTCTTCATCAATCAGTGGCAGATCAACCATGATCCAGAGATTTGG 1499
D T S L N G Y F I P K D T C V F I N Q W Q I N H D P E I W 426
AAAGATCCATCTTCCTTCAACCCAGATCGCTTCCTGAGTGCTGATGGCACTGAGGTCAACAAGATGGAAGGGGAGAAGATATCAATA 1566
K D P S S F N P D R F L S A D G T E V N K M E G E K I S I 455
TTTGGCATGGGGAAGCGCCGC**TGC**ATCGGCGAGGTCATTGCACGAAATGAAGTCTACCTCTTCTTGGCAATCATTGTCCAGAAGCTG 1653
F G M G K R R C I G E V I A R N E V Y L F L A I I V Q K L 484
CAATTCCATGCGATGCCTGGAAAGCCACTGGACATGACACCAGAGTACGGTCTCACAATGAAGCACAAACGTTGCCACCTGAGAGCC 1740
Q F H A M P G K P L D M T P E Y G L T M K H K R C H L R A 513
ACAATGCGAGTGAGAAATGAGCAG**TAA**AGCTATTCAGAATGTACAATGTATGACTCACTAAGTCGTATAAGTTGACTCTAACAATGC 1827
T M R V R N E Q 521
ATGGGTCACCTTCAAGTCAAGAGTAAGTGAAACAAGCATCTTTCTCAGAATGTAAGGCACCGAATGCCTAATTTGATTCACAGGCTT 1914
ACTGGCATTGAAGCAAATCAATGAATGTTGCTTGCTGCAGATTGTCAGAGATGACTATGTTTGTGTGAACATATCCCTGTCATGTTT 2001
TTGGTTCTCTGTGAGCTCTTCCTGCACATGATTGGTGCTTCTCAGTGAAGTATAGGAGAGATACTTGGTTCTCCTGAGTTGTGTAGT 2088
AAACTAAACTGTTCCCATTAAGAAGTTATAAATTGCTCAGAGATTAAATTACCATGTTCAACTTTGGGGCAAGCAGTATGTTTTACT 2175
GGATGTTTTTCTTCTGTGACACAGGACTCAGAACTGAACAGCTAATACCTTTTGATACC**ATTTA**CACTTGTAAGCTAAATATTTTTA 2262
TTCCATAATATAATGTGGATGTAAGACACTGAAGCTATATTTGTATCCCAAAATGTGATTCTGCTGTATATCAAATTCTTTGTATTT 2349
TATAAAGCCATTTTCATGTGCCTAGCGTTTGTATATATCTGAGGTGAAATAGTTTAGCTGTTT**ATTTA**CATGGAATATAAACCCACG 2436
AAAAGAGCTCCAGTGAGTTTTTGGAATATATTGGAAGGCTAAACAATTTTGTATTTC**TATA**TATAACGTATAAATGATTGTATAACA 2523
TGTATGGG**CGTG**TGATTGGAGCATCCTTTAATCAGCCACACAAAT**AAAATAAAA**TATGTTGTAAAAAAAAAAAAAAAAAAAAAAA 2608

Figure S1. Nucleotide and deduced amino acid (below the former) sequences of Atlantic croaker cytochrome P450 1A gene. The start codon, heme-binding cysteine codon (position 463), stop codon (TAA), ATTTA (AUUUA) pentanucleotide sequences, TATA box, and putative poly-adenylation signal (AAAATAAAA) are all underlined and boldfaced. GenBank accession number JQ622220.
