# Supplementary material for: Effects of Hypoxia Exposure on Hepatic Cytochrome P450 1A (CYP1A) Expression in Atlantic Croaker: Molecular Mechanisms of CYP1A Down-Regulation
Source: PLoS One. 2012 Jul 16;7(7):e40825. doi: 10.1371/journal.pone.0040825 (PMC3397942; doi:10.1371/journal.pone.0040825)
Supplement: Figure S2 — Amino acid sequence of CYP1A protein. Alignment of the full-length amino acid sequence of Atlantic croaker cytochrome P450 1A (CYP1A) with CYP1A-related protein of other vertebrates (A). Amino acid sequence identity (%) between croaker CYP1A and those of other vertebrates (B). Dots indicate residues that are identical to croaker CYP1A1. Dashes indicate gaps introduced to facilitate alignment. GenBank accession numbers for the sequences used are as follows: croaker (JQ622220), seabass (DLU78316), scup (U14162), rainbow trout (T.) (M21310), rainbow trout (M21310), zebrafish (AF210727), whale (AB231891) and human (K03191). The substrate recognition site (SRS) and heme-binding region (HBR) are underlines. (PDF) [file pone.0040825.s002.pdf]

A

```
                                                    SRS-1
Croaker     MVLMILPFIGSVSVSESLVAITVLCVVYLALRCFHTEIPEGLRRLPGPKPFPLIGNVLEVGSKPYLSLTAMSKRYGDVFQIQVGMRPVIV   90
Seabass     ...................L.TV.....I.KF.R.......H.......L..........N...................I.....V.   90
Scup        .......V........G...MITM..A..I..L.R.......LQ......L.I........RN..................I.....V.   90
Rainbow T.  .......II....A..G...MVT..L.YMIMKYM........K.......L.I.......HNN.H.......E...S.....I.....V.   90
Zebrafish   .A.T...IL.PI.........ITI.....LM.LNR.K..D..QK......L.I...V..I.NN.H........C..P.....I.....V.   90
Whale        MFSVFGLSIPI.AT.L.L.SATF...FWVV.AWQPRV.K..KSP...WSW....H..TL.KS.H.A.SRL.Q.....L..RI.CT..L.   89
Human            ML.PI.M.AT.F.L.SVIF...FWVM.ASRPQV.K..KNP...WGW....HM.TL.KN.H.A.SR..QQ....L..RI.ST..V.   85

Croaker     LSGTDTVRQALIKQGEEFAGRPDLYSFRFINDGKSLAFSTDQAGVWRARRKLAYSALRSFSSLEGTTPEYSCMLEEHICKEGEYLIKQLN  180
Seabass     ...SE.........D...........................................G........V................  180
Scup        ...SE.........DX.....................................AT..........A....VS..A...V...H  180
Rainbow T.  ...SE..........D.........K............K..........M......AT..........A....V.......V...T  180
Zebrafish   ...N.VI....L......S...E...TK.............V..........LN...T..TVQ.KS.K...A.....SN..L..VQR.H  180
Whale       ...L..I....VR..DD.K........TLVA..Q.MT.NP.SGP..A...R..QN..K...IASDPASSS..Y....VS..S....GKFQ  179
Human       ...L..I....VR..DD.K......T.TL.SN.Q.MS.SP.SGP..A...R..QNG.K...IASDPASST..Y....VS..A.V..ST.Q  175

                                                            SRS-2                    SRS-3
Croaker     NVMKADGSFDPFRHIVVSVANVICGMCFGRRYDHNDQELLSLVNLSDEFGQVVGSGNPADFIPILQYLPSSTMKTFMDINVRFNTFVQKI  270
Seabass     T.....................................................................V..F...T...K.....A...K.....  270
Scup        T..E..........................H....N...........A...............T...K.LN..D.........  270
Rainbow T.  S..DVS.......................S.D.....G...M..............S...LR...NR...R.....D.......N.  270
Zebrafish   S........................I.....HS.D.D..VR...M.....KI...........F.RI...T...K.L...E..SK.MKRL  269
Whale       EL.AGS.R...Y.YV.........A.........ES.V...V.G..N...A.AA...........R...NTALDD.K.L.R..YI.M..M  269
Human       EL.AGP.H.N.Y.YV....T....AI.........H.......N.NNN..E.......E.....R...NPSLNA.K.L.EK.YS.M..M  265

                                                         SRS-4
Croaker     VSEHYANYNKDNIRDITDSLIDHCEDRKLDENSNVQMSDEKVVGIVNDLFGAGFDTISTALSWSVMYLVAHPEMQERLYQELKDTVGLDR  360
Seabass     .....TT.D.............................I...........................Y..IE........EN.....  360
Scup        .....TTFD.............................I...........................Y..I.......MNE...P..  360
Rainbow T.  I....ES.D....................A.I.V....I....................A.V....Y..I....H....EK..MI.  360
Zebrafish   .M...DTFD............N...........L.V....I....................A.V...HY..V....QR..DEKI.K..  360
Whale       LK...KTFE.GR.........E..QGKR....A.I.L....I.NV.M.........VT..I...L....TS.SV.KKIQE..DTVI.SA.  359
Human       .K...KTFE.GH.........E..QEKQ....A.V.L....IINI.L.........VT..I...L....MN.RV.RKIQE..DTVI.RS.  355

                                SRS-5
Croaker     SPRLSDRPSLPFLDAFILEIFRHSSFLPFTIPHCTSKDTSLNGYFIPKDTCVFINQWQINHDPEIWKDPSSFNPDRFLSADGTEVNKMEG  450
Seabass     T.L.C...N....E..............................................L.............T....L..L..  450
Scup        T.C...K.K....E.....T..........................................A.L.............NA.......L..  450
Rainbow T.  T.....KIN..L.E....................I.....................V....DL..E................L..L..  450
Zebrafish   T.L....AN..L.ES.................................V....V.....L.....S.I.....TA....L..L..  450
Whale       Q.......R..Y.E.....T.............S.TR......F....GR...V........QKL.D...A.W.E...TA...-I..ALS  448
Human       R.....RSH..YME.....T......V......S.TT....K.F....GR...V........QKL.VN..E.L.E...TP..A-ID.VLS  444

                   HBR                                  SRS-6
Croaker     EKISIFGMGKRRCIGEVIARNEVYLFLAIIVQKLQFHAMPGKPLDMTPEYGLTMKHKRC-HLRATMRVRNEQ     521
Seabass     ..VMV..L........................H.KTL..E................-.......ASE        519
Scup        ..MMV...............S........L..N.R..S...E................-Q...A..A...E    521
Rainbow T.  ..VLV...D.......A.G....F.....LL.R.R.QEK..H............H...-Q.K.S..PWGQEE   522
Zebrafish   ..VLV..L.......S.G.A.......LL.R.K.TG...EM...............-L..V.PQPGF       519
Whale       ..VIL..L...K....T...W........LL.QVE.RVT..VKV....V.......AH.E.FQ.HM.S       516
Human       ..VI.......K....TV..W........LL.RVE.SVPL.VKV....I.......AC.E.FQMQL.S       512
```

B

Sequence identity	
Seabass	89
Scup	85
Rainbow trout	80
Zebrafish	73
Whale	56
Human	55

Figure S2. Amino acid sequence of CYP1A protein. Alignment of the full-length amino acid sequence of Atlantic croaker cytochrome P450 1A (CYP1A) with CYP1A-related protein of other vertebrates (A). Amino acid sequence identity (%) between croaker CYP1A and those of other vertebrates (B). Dots indicate residues that are identical to croaker CYP1A. Dashes indicate gaps introduced to facilitate alignment. GenBank accession numbers for the sequences used are as follows: croaker (JQ622220), seabass (DLU78316), scup (U14162), rainbow trout (T.) (M21310), rainbow trout (M21310), zebrafish (AF210727), whale (AB231891) and human (K03191). The substrate recognition site (SRS) and heme-binding region (HBR) are underlines.
