# Supplementary material for: Effects of Hypoxia Exposure on Hepatic Cytochrome P450 1A (CYP1A) Expression in Atlantic Croaker: Molecular Mechanisms of CYP1A Down-Regulation
Source: PLoS One. 2012 Jul 16;7(7):e40825. doi: 10.1371/journal.pone.0040825 (PMC3397942; doi:10.1371/journal.pone.0040825)
Supplement: Figure S3 — Molecular phylogeny of CYP1A protein. The phylogenetic tree was constructed using the Neighbour-Joining method (1000 bootstrap). Bootstrap values are shown at the branch points. Scale bar indicates the number of changes inferred as having occurred along each branch. GenBank accession numbers for European seabass (CAB63650), Japanese seabass (ADC35580), tiger bass (ABZ88704), Atlantic croaker (JQ622220), large yellow croaker (ACT64126), four-eye butter flyfish (Q92039), scup (U14162), gilthead seabream (O42457), sand steenbras (AAK69390), black porgy (ABI54450), Japanese flounder (ABO38813), marbled flounder (BAC87834), winter flounder (ADV36120), common dab (O42430), European flounder (Q9YH64), European plaice (Q92100), killifish (AAD01809), mangrove rivulus (AAQ16634), Japanese medaka (AAP48792), flathead mullet (ABZ88706), golden gray mullet (O42231), thicklip gray mullet (ABD95933), leaping mullet (Q9W683), pufferfish (ABV24057), spotted green pufferfish (CAG03127), three spined stickleback (ADO15701), spotted snakehead (ACL31529), Atlantic salmon (AAM00254), brook trout (AAQ10899), lake trout (AAQ10900), rainbow trout (AAD14035), Japanese eel (BAA88241), European eel (AAL99904), yellow catfish (EF584508), rare minnow (ABV01348), goldfish (ABF60890), zebrafish (NP_571954), African clawed frog (BAA37079), tropical clawed frog (AAI35261), guinea pig (NP_001166411), mouse (Y00071), whale (AB231891) and human (K03191). (PDF) [file pone.0040825.s003.pdf]

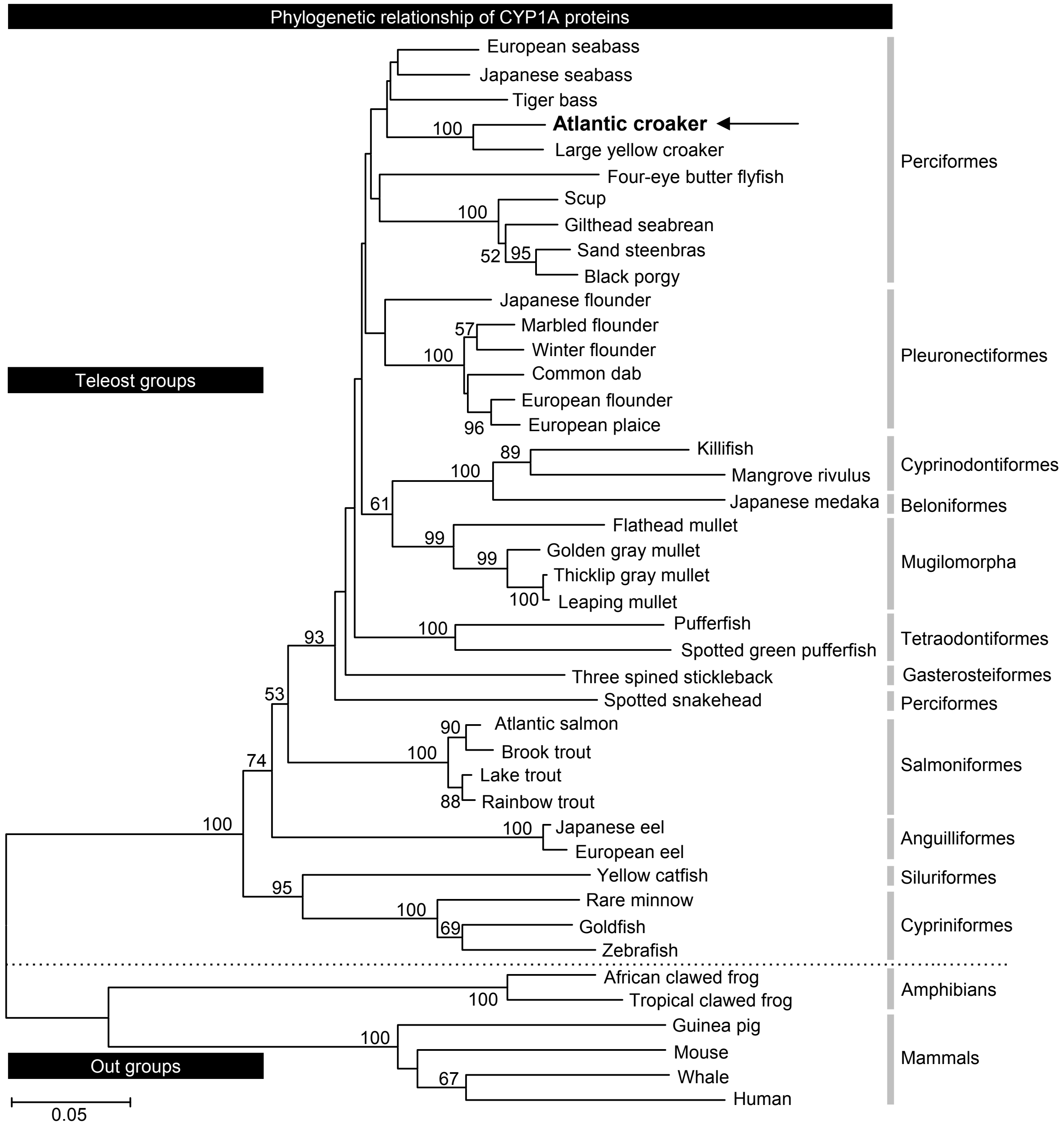

Figure S3. Molecular phylogeny of CYP1A protein. The phylogenetic tree was constructed using the Neighbour-Joining method (1000 bootstrap). Bootstrap values are shown at the branch points. Scale bar indicates the number of changes inferred as having occurred along each branch. GenBank accession numbers for European seabass (CAB63650), Japanese seabass (ADC35580), tiger bass (ABZ88704), Atlantic croaker (JQ622220), large yellow croaker (ACT64126), four-eye butter flyfish (Q92039), scup (U14162), gilthead seabream (O42457), sand steenbras (AAK69390), black porgy (ABI54450), Japanese flounder (ABO38813), marbled flounder (BAC87834), winter flounder (ADV36120), common dab (O42430), European flounder (Q9YH64), European plaice (Q92100), killifish (AAD01809), mangrove rivulus (AAQ16634), Japanese medaka (AAP48792), flathead mullet (ABZ88706), golden gray mullet (O42231), thicklip gray mullet (ABD95933), leaping mullet (Q9W683), pufferfish (ABV24057), spotted green pufferfish (CAG03127), three spined stickleback (ADO15701), spotted snakehead (ACL31529), Atlantic salmon (AAM00254), brook trout (AAQ10899), lake trout (AAQ10900), rainbow trout (AAD14035), Japanese eel (BAA88241), European eel (AAL99904), yellow catfish (EF584508), rare minnow (ABV01348), goldfish (ABF60890), zebrafish (NP_571954), African clawed frog (BAA37079), tropical clawed frog (AAI35261), guinea pig (NP_001166411), mouse (Y00071), whale (AB231891) and human (K03191).
