# Supplementary material for: Effects of Hypoxia Exposure on Hepatic Cytochrome P450 1A (CYP1A) Expression in Atlantic Croaker: Molecular Mechanisms of CYP1A Down-Regulation
Source: PLoS One. 2012 Jul 16;7(7):e40825. doi: 10.1371/journal.pone.0040825 (PMC3397942; doi:10.1371/journal.pone.0040825)
Supplement: Figure S4 — Nucleotide and deduced amino acid (below the former) sequences of Atlantic croaker interleukin-1β cDNA. Putative N-glycosylation site is underlined. GenBank accession number JQ622219. (PDF) [file pone.0040825.s004.pdf]

```
  GGAATGCAACGTGAGCGAGATGTGGGGCCACAAGATGCCCAGGGGACTGGACTTGGAA    58
    E  C  N  V  S  E  M  W  G  H  K  M  P  R  G  L  D  L  E     19
GTTACCCATCATCCACACACAATGAAGCATGTGGTCAACCTCATCATTGCCATTGAGAGG   118
 V  T  H  H  P  H  T  M  K  H  V  V  N  L  I  I  A  I  E  R     39
TTAAAGGGCAGAGGGTCAGACTTACTGCTGAATGCCACCGAGTTCAGAGATGAAAACTTG   178
 L  K  G  R  G  S  D  L  L  L  N  A  T  E  F  R  D  E  N  L     59
CTCAACTTCGTGCTGGAGAACATAGTGGAAGAGCGAATTGTGTTCGGGTGTAATGCAGCT   238
 L  N  F  V  L  E  N  I  V  E  E  R  I  V  F  G  C  N  A  A     79
GCACCAGTTCAGTACACAAAGACGGAGGATTACCAGTGCAGCGTGACCGACAGTGAGAAG   298
 A  P  V  Q  Y  T  K  T  E  D  Y  Q  C  S  V  T  D  S  E  K     99
AGGAGCTTAGTTCTGGTCCGAAACAGCATGGAACTCCATGCTGTGATGCTGCAGGGAGGC   358
 R  S  L  V  L  V  R  N  S  M  E  L  H  A  V  M  L  Q  G  G    119
GCTGAGAATCGCAAAGTTCACTTGAACATGGCGACCTACGTACACCCTGCACCCAGTGCG   418
 A  E  N  R  K  V  H  L  N  M  A  T  Y  V  H  P  A  P  S  A    139
GAGGCCCAGACTGTGGCTCTGGGCATCAAGGGCACAAATTTCTACCTCTCTTGCCATAAG   478
 E  A  Q  T  V  A  L  G  I  K  G  T  N  F  Y  L  S  C  H  K    159
GATGGGAACAACAAGCCATCCTTGCACCTGGAGACAGTGGAAAATTCAACCAACCTGGCA   538
 D  G  N  N  K  P  S  L  H  L  E  T  V  E  N  S  T  N  L  A    179
AGTATCAGCTCGGACAGCGAGATGGTTCGATTTCTGTTCTACAAACAAGACACTGGGCTG   598
 S  I  S  S  D  S  E  M  V  R  F  L  F  Y  K  Q  D  T  G  L    199
AACCTCAGTACCCTTGTGTCTGTCCCCTACAACGACTGGTACAT                   642
 N  L  S  T  L  V  S  V  P  Y  N  D  W  Y                      213
```

**Figure S4. Nucleotide and deduced amino acid (below the former) sequences of Atlantic croaker interleukin-1β cDNA.** Putative *N*-glycosylation site is underlined. GenBank accession number JQ622219.
